# Growth inhibition and differentiation of human breast cancer cells by the PAFR antagonist WEB-2086

**DOI:** 10.1038/sj.bjc.6603156

**Published:** 2006-05-23

**Authors:** C Cellai, A Laurenzana, A M Vannucchi, R Caporale, M Paglierani, S Di Lollo, A Pancrazzi, F Paoletti

**Affiliations:** 1 Department of Experimental Pathology and Oncology, School of Medicine, University of Florence, Viale G. B. Morgagni 50, 50134 Florence, Italy; 2 Department of Haematology, University of Florence, Viale Pieraccini 17, 50139 Florence, Italy; 3 Department of Human Pathology and Oncology, School of Medicine, University of Florence, Viale Pieraccini 17, 50139 Florence, Italy

**Keywords:** breast cancer, differentiation, growth arrest, WEB-2086, MCF-7, MDA-MB-231

## Abstract

WEB-2086 – an antagonist of platelet-activating factor receptor (PAFR) with known anti-inflammatory, antiangiogenic and antileukaemic properties – also proved to inhibit the proliferation in human solid tumour cell lines of different histology, and with much higher efficacy than in normal fibroblasts. A detailed analysis of WEB-2086 anticancer activity was then performed focusing on breast adenocarcinoma MCF-7 and MDA-MB-231 cells. WEB-2086-treated cells, either expressing (MCF-7) or unexpressing (MDA-MB-231) the oestrogen receptor (ER)*α*, underwent a dose-dependent growth arrest (IC_50_=0.65±0.09 and 0.41±0.07 mM, respectively) and accumulation in G_0_–G_1_ phase. WEB-2086 also induced morphological and functional changes typical of mature mammary phenotype including (i) cell enlargement and massive neutral lipid deposition (best accomplished in MCF-7 cells); (ii) decrease in motility and active cathepsin D levels (mainly observed in highly invasive MDA-MB-231 cells). The expression of ER*α* was neither increased nor reactivated in treated MCF-7 or MDA-MB-231 cells, respectively. WEB-2086-induced differentiation in breast cancer cells involved the upregulation of PTEN, a key tumour suppressor protein opposing tumorigenesis, and was apparently independent of p53, PAFR, peripheral benzodiazepine receptor and ER*α* status. Overall, WEB-2086 can be proposed as an effective antiproliferative and differentiative agent with interesting translational opportunities to treat breast cancers in support to conventional chemotherapy.

Differentation-inducing therapy found its best clinical application by using all-*trans*-retinoic acid (ATRA) (Warrell *et al*, 1991) and STI571 (Druker, 2002) to cure successfully acute promyelocytic leukaemia (APL) and chronic myelogenous leukaemia, respectively. Such excellent effects, however, were not reproduced in other haematological and, particularly, in solid tumours, which, therefore, represent a real challenge for differentiative agents.

We showed previously that WEB-2086 – an antagonist of platelet-activating factor receptor (PAFR) with known anti-inflammatory properties (Ishii and Shimizu, 2000) – could also induce erythroleukaemia cell maturation (Cellai *et al*, 2002), enhance ATRA differentiation potential, trigger apoptosis in ATRA-sensitive and -resistant APL cell lines, and in blasts from patients with APL (Laurenzana *et al*, 2005). These results prompted us to investigate WEB-2086 effects also in solid tumour cells. We examined different cell lines from human breast adenocarcinoma, colon adenocarcinoma, hepatocarcinoma, fibrosarcoma, and neuroblastoma and found that the drug induced growth arrest in all types of cancer cells and with much higher efficacy than in normal cells.

WEB-2086 activity was then systematically analysed in MCF-7 and MDA-MB-231 breast adenocarcinoma cell lines. MCF-7 are low-migrating cells expressing oestrogen receptor (ER)*α* while MDA-MB-231 are ER*α*-negative cells with high mobility and invasive potential (Bussolati *et al*, 2000). These models have been widely used to test *in vitro* differentiation activity of agents like Na-butyrate and hexamethylene bisacetamide (HMBA) (Guilbaud *et al*, 1990), and retinoids including ATRA (Wang *et al*, 2001). More recently, emphasis was given to histone deacetylase inhibitors (HDACi) such as suberoylanilide hydroxamic acid (SAHA) and trichostatin A (TSA) which induce differentiation in breast cancer cells *in vitro* (Yang *et al*, 2000; Munster *et al*, 2001; Jang *et al*, 2004) by altering their transcriptional programs. However, the clinical use of HADCi including valproic acid to treat breast cancer is severely limited by the drug-mediated increase of ER*α* expression and tissue sensitization to tumour promotion activity of oestrogens (Akhmedkhanov *et al*, 2001; Graziani *et al*, 2003).

In this study we report that WEB-2086 exerted a broad cytostatic effect in cell lines from solid tumours of different histology. Moreover, with respect to particularly MCF-7 and MDA-MB-231 breast adenocarcinoma cells, WEB-2086 could re-establish cell growth and migration control regardless of ER*α* status, and restore also some traits of mature mammary phenotype without enhancing and/or reactivating ER*α* expression. All these properties point to WEB-2086 as a novel differentiation agent with a low-toxic profile *in vivo* (Adamus *et al*, 1989) and with interesting therapeutic potential for treating breast cancers in support to conventional cytotoxics.

## MATERIALS AND METHODS

### Cells, culture conditions and reagents

Media used (Bio-Whittaker Europe, Verviers, Belgium) were IMDM for MCF-7 and Hep-G2; DMEM for MDA-MB-231, HT-1080 and dermal fibroblasts; RPMI for SH-SY5Y (plus 1% non-essential AA) and HCT-8 (plus 1 mM Na-pyruvate). Cells were propagated in the presence of 10–15% foetal bovine serum (FBS, EuroClone, Life Science Division, Milan, Italy) and 2 mM L-glutamine at 37°C in 5% CO_2_ humidified atmosphere. Cells were harvested with a trypsin-EDTA solution (Bio-Whittaker Europe), plated (1.5 × 10^4^ ml^−1^) and left for 6 h before any treatment. WEB-2086 (Casals-Stenzel *et al*, 1987) (Boehringer Ingelheim Pharma KG, Biberach, Germany), was dissolved in dimethyl sulfoxide (DMSO; Sigma-Aldrich, St Louis, MO, USA); the stock solution (260 mM) was stored in the dark at room temperature. HMBA, Na-butyrate and ATRA were from Sigma-Aldrich; other chemicals were reagent grade. Cells treated with increasing WEB-2086 for 72 h were harvested and counted in a Bürker chamber to assess the drug concentration necessary to reduce cell density by 50% as compared to control (IC_50_ value).

### Flow cytometry

Cell cycle distribution was determined by the propidium iodide (PI)-hypotonic citrate method with a FACScan instrument (Becton-Dickinson, San Jose, CA, USA) (Paoletti *et al*, 1996).

The number of cell replications was assessed with the aid of carboxyfluorescein diacetate succinimidyl ester (CFSE; Molecular Probes, Eugene, OR, USA), which covalently binds cell components to yield a fluorescence that was divided equally between daughter cells at each division (Lyons *et al*, 2001). Cells were starved for 14 h with the medium containing 0.5% FBS, then harvested and labelled with CFSE following the manufacturer's instruction. Labelled cells (day 0) were used to measure initial levels of fluorescence and then seeded (2 × 10^4^ cells ml^−1^) with or without either WEB-2086 (0.5–1.5 mM) or DMSO as the vehicle (0.19–0.57%) to calculate replication number after 5 days of treatment (software: ModFit LT for Macintosh, Proliferation Protocol, Verity Software House Inc., Topsham, ME, USA).

### Cell morphology, apoptosis and neutral lipid determination

Morphological changes were microscopically examined either by phase contrast or ethanol-fixed cells stained with haematoxylin-eosin using a Digital Camera System Leica DC 200 (Leica Microsystems, Inc. Bannockburn, IL, USA). Apoptosis was revealed by the presence of nuclear fragmentation in stained cells and by the DNA ladder assay as reported previously (Laurenzana *et al*, 2005).

Neutral lipids were determined (i) histochemically (Constantinou *et al*, 1998) on cell monolayers which were quickly fixed with −20°C methanol, stained with Oil Red O (Sigma-Aldrich) and counterstained with haematoxylin; or (ii) spectrophotometrically (Cary 50 Scan, Varian, Victoria, Australia) at 510 nm by recording absorbance of cell-bound Oil Red O following extraction with isopropanol (Wang *et al*, 2001).

### Reverse transcription–polymerase chain reaction

Total RNA was prepared with TRIzol (Invitrogen, Carlsbad, CA, USA), following the manufacturer's instructions. Samples (1 *μ*g each) were reverse-transcribed and then amplified by PCR with the following primers. For ER*α*, 5′-CAAGCCCGCTCATGATCA-3′ and 5′-CACCATGCCCTCTACACA-3′ (388 bp); for peripheral benzodiazepine receptor (PBR), 5′-CACGCTCTACTCAG-CCATGG-3′ and 5′-GCAGTAGTTGAGTGTGGTCGC-3′ (298 bp); for PAFR, 5′-ACCAACACAGTGCCCGACAGTGCT-3′ and 5′-GGGTGACCTGATGTGCATCA-TTAAT-3′ (363 bp); for *β*_2_-microglobulin, 5′-CTCGCGCTACTCTCT-CTTTCT-3′ and 5′-ACATGGAGACAGCACTCAAAG-3′ (514 bp). Reverse transcription-polymerase chain reaction (RT–PCR) products were analysed as described previously (Cellai *et al*, 2002).

### Cell migration

Cell mobility was assessed in Boyden's chambers using a polyvinyl-pyrrolidone-free polycarbonate filter (8 *μ*m pore size) between the two chambers (Alessandri *et al*, 1986). After a 6-h incubation at 37°C, cells (2.5 × 10^4^, 0.2 ml^−1^) on the upper surface of filters were scraped; filters, were then fixed in methanol, stained with Wright-Giemsa and photographed as above.

### SDS–PAGE and Western blotting

Harvested cells were resuspended in the lysis buffer (Laurenzana *et al*, 2005) and separated on 12.5% SDS–PAGE. Western-blot membranes were probed using primary antibodies against cathepsin D, p53, PTEN, and *α*-tubulin (Santa Cruz Biotech, Santa Cruz, CA, USA) and ER*α* (Ventana Medical Systems, Tucson, AZ, USA). Peroxidase-conjugated secondary antibodies (Santa Cruz Biotech) and the ECL procedure on Hyperfilm ECL (Amersham Pharmacia Biotech) were used for development.

### Statistical analysis

All experiments were independently done at least three times. All data were statistically analysed by Student's *t*-test.

## RESULTS

### WEB-2086 inhibited proliferation in six different solid tumour cell lines

Human tumour cells from breast adenocarcinoma (MCF-7 and MDA-MB-231), ileocecal adenocarcinoma (HCT-8), hepatocarcinoma (Hep-G2), fibrosarcoma (HT-1080), and neuroblastoma (SH-SY5Y) were cultured with increasing WEB-2086 concentrations for 3 days. IC_50_ values of solid tumour cells were within the range of 0.38–0.83 mM WEB-2086 (mean value ∼0.61 mM) (Table 1). HT-1080 and HCT-8 were the more and the less drug-responsive cells, respectively, while normal human fibroblasts used as control yielded a mean IC_50_ value >4 mM. These data indicated that solid tumour cells, irrespective of their histogenesis, were all especially sensitive to WEB-2086-induced cytostasis thus inferring that a common pathway might be involved. WEB-2086 effects have been then analysed further in breast adenocarcinoma cells: MCF-7 which are hypotetraploid, ER*α*-positive and low-mobility cells expressing the wild-type p53 form; and MDA-MB-231 which are hypotriploid, ER*α*-negative and highly invasive cells carrying a mutated and inactive p53 form.

### WEB-2086 induced growth arrest and differentiation in MCF-7 cells

**Figure 1:**
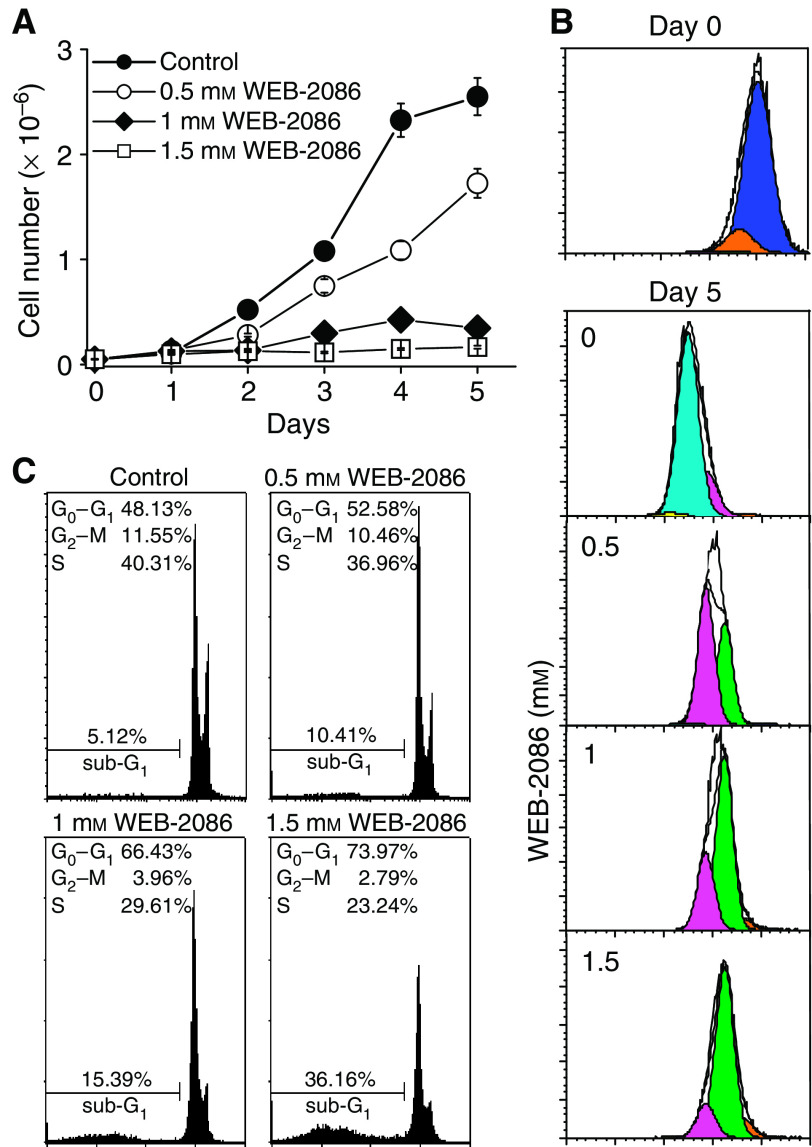
WEB-2086 effects on MCF-7 cells. (**A**) Cultures treated for 5 days with increasing (0–1.5 mM) drug were monitored for changes in cell density. (**B**) Cells were labelled with CFSE and immediately examined to be used as the starting population (top panel, day 0, 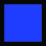). After a 5-day incubation with 0–1.5 mM WEB-2086, the generation numbers were calculated (bottom panel) and indicated by the listed colours: first (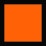), second (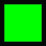), third (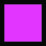), fourth (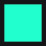), fifth (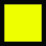). (**C**) Flow cytometry profiles of PI-stained MCF-7 cells incubated for 2 days with 0–1.5 mM WEB-2086. Percentages of cells in the different phases of cell cycle were reported. Experiments carried out with DMSO as the vehicle (up to 0.57%) showed negligible interference with cell growth, replication rounds and cell cycle (data not shown).

WEB-2086-treated MCF-7 cells underwent a striking inhibition of growth that was virtually abolished at ⩾1 mM drug (Figure 1A). WEB-2086 effects on MCF-7 replicative potential was evaluated by tracking subsequent rounds of cell division with the fluorescent dye CFSE (see Materials and methods) after a 5-day incubation of cultures with or without increasing WEB-2086 concentrations (Figure 1B and legend). Approximately 75% of untreated MCF-7 cells approached the fourth generation and 23% reached the third generation. Conversely, in MCF-7 cultures incubated with 1 or 1.5 mM WEB-2086, the predominant fraction (67 and 92%, respectively) was represented by cells arrested after two replications only.

Cytofluorimetric analysis of cell cycle (Figure 1C) showed that a 2-day treatment led to a dose-dependent MCF-7 accumulation in G_0_–G_1_ phase to reach approximately 66 and 74% of population at 1 and 1.5 mM drug, respectively. Concomitantly, there was a decrease in G_2_-M/S cell fraction and a moderate increase in the sub-G_1_ fraction over basal levels. However, DNA ladder assay and morphology of treated cells indicated that apoptosis involved only a minor portion of the population (data not shown).

**Figure 2:**
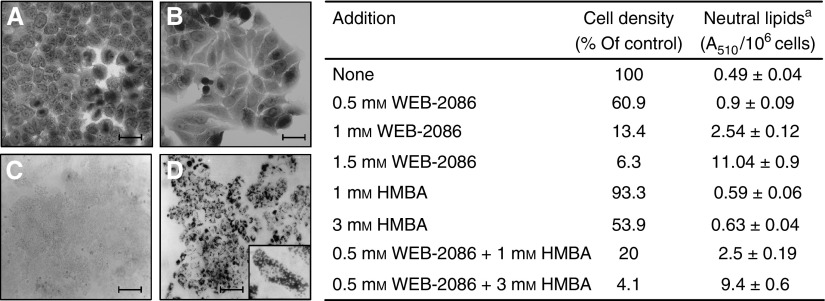
Morphological and functional differentiation of MCF-7 by WEB-2086 and its synergy with HMBA. *Left panel*: Changes in morphology were observed (× 40) following haematoxylin-eosin staining of cells grown onto glass-coverslips in the absence (**A**) or in the presence (**B**) of 1 mM WEB-2086 for 5 days. Neutral lipids were revealed histochemically by Oil Red O staining of MCF-7 (× 10) incubated for 7 days without (**C**) or with 1 mM WEB-2086 (**D**) and insert (× 40). Magnification bars **A**–**B**=25 *μ*m, **C**–**D**=100 *μ*m. *Right panel*: Neutral lipid content (±s.d.) in cultures treated for 7 days with increasing WEB-2086 (0–1.5 mM) and/or HMBA (1–3 mM) concentrations was determined by absorbance at A_510_/10^6^ cells in isopropanol extracts of Oil Red O-stained cells. ^a^Spectophotometric Assay (Wang *et al*, 2001).

MCF-7 incubated with 1 mM WEB-2086 showed features that were reminiscent of mature mammary phenotype including an increase in cell size and eosinophilia, a reduced nuclear/cytoplasmic ratio, and a shift from a round to columnar shape to yield adenoma-like clusters where a central lumen was often recognized (Figure 2 left, B *vs* A). Red Oil O staining of treated MCF-7 cells revealed a massive accumulation of neutral lipids, which are an important milk component and the most typical trait of mature epithelial mammary cells (Figure 2 left, D and insert, *vs* C).

Cell density and neutral lipid levels were determined in MCF-7 incubated with increasing amounts of either WEB-2086 or HMBA given alone or in combination (Figure 2, right). WEB-2086 caused a marked dose-dependent decrease in cell density and increase in neutral lipid content of approximately twofold, fivefold and 22-fold over control levels after a 7-day incubation with 0.5, 1 and 1.5 mM drug, respectively. HMBA was much less efficient than WEB-2086 in inhibiting cell growth and inducing neutral lipid accumulation. However, the two inducers acted synergistically. The combinations of 0.5 mM WEB-2086 with either 1 or 3 mM HMBA inhibited MCF-7 cell growth and increased neutral lipid levels with higher efficacy than the two drugs alone. No synergy was observed between WEB-2086 and either Na-butyrate or ATRA (data not shown).

### Response of MDA-MB-231 cells to WEB-2086

**Figure 3:**
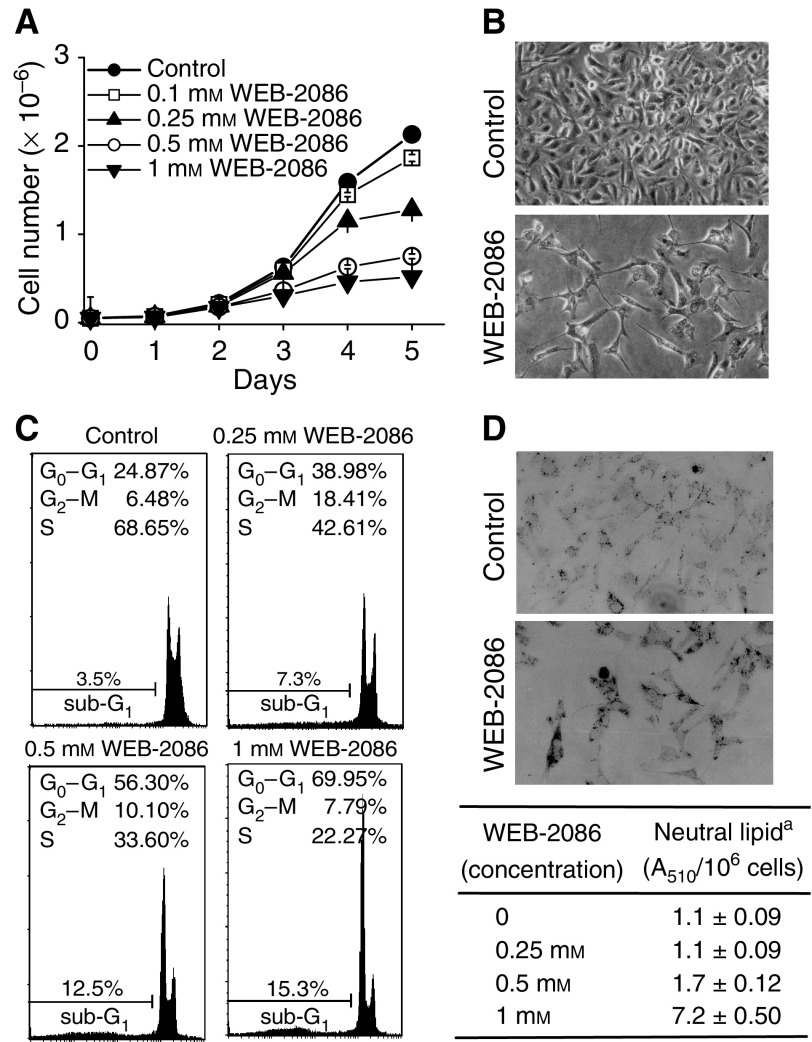
WEB-2086 effects on cell growth and differentiation of MDA-MB-231 cells. (**A**) Cell density was monitored in cultures treated for 5 days with increasing drug (0–1 mM). (**B**) Morphological changes in MDA-MB-231 untreated (control) or treated with 0.5 mM WEB-2086 for 5 days (× 20); magnification bars=50 *μ*m. (**C**) Flow cytometry profiles of PI-stained MDA-MB-231 cells incubated for 2 days with WEB-2086 (0–1 mM); percentages of cells in different phases of cell cycle were reported. (**D**) *Top panel*: Histochemistry of neutral lipids was performed by the Oil Red O staining of MDA-MB-231 cultured for 14 days without (control) or with 0.5 mM WEB-2086 (× 10); *bottom panel*: spectrophotometric evaluation of neutral lipid levels (±s.d.) in cultures incubated for 14 days with increasing WEB-2086 as determined in Figure 2.

ER*α*-negative MDA-MB-231 cells were approximately 40% more sensitive than ER*α*-positive MCF-7 cells to drug-induced inhibition of cell growth (Figure 3A and Table 1). Treated MDA-MB-231 turned into scanty elongated and moderately enlarged fibroblast-like cells with no tendency to form bundles (Figure 3B). Cell cycle analyses showed that approximately 56 and 70% of MDA-MB-231 treated for 2 days with 0.5 and 1 mM WEB-2086, respectively, were arrested in G_0_–G_1_ phase; concomitantly, the G_2_-M/S cell fraction decreased while the sub-G_1_ fraction slightly increased (Figure 3C).

Neutral lipids were already detectable in untreated MDA-MB-231 cells but increased markedly upon WEB-2086 addition (Figure 3D) though at half the rate of MCF-7 cells.

### WEB-2086 reduced breast cancer cell mobility and active cathepsin D levels

**Figure 4:**
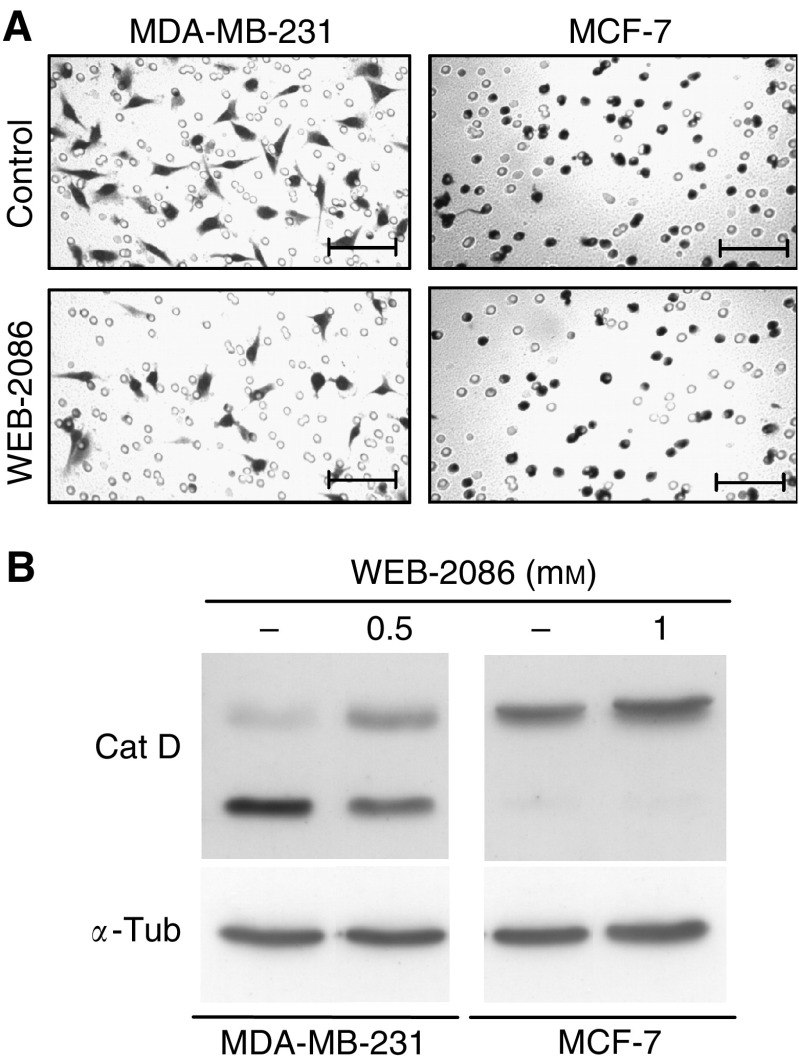
WEB-2086-mediated effects on cell invasive potential. (**A**) MDA-MB-231 and MCF-7 were previously incubated for 5 days without (control) or with WEB-2086 (0.5 and 1 mM, respectively) and then transferred to the Boyden's chamber for 6 h. Cells on the lower side of the filter were then stained and counted to determine migration activity; magnification bars=100 *μ*m. (**B**) Western-blot analysis of cathepsin D in MDA-MB-231 and MCF-7 cells incubated for 5 days with 0.5 and 1 mM of WEB-2086, respectively; *α*-tubulin was used as the loading control.

MDA-MB-231, unlike MCF-7, are cells with high mobility and have been used, therefore, to monitor WEB-2086 effects on cell migration and cathepsin D levels which associate closely with breast cancer invasiveness (Rochefort *et al*, 2000). Results of Boyden's chamber experiments (Figure 4A) showed that a 5-day incubation of MDA-MB-231 with 0.5 mM WEB-2086 decreased cell migration of approximately 75% while the low-migrating activity of MCF-7 was virtually unaffected by 1 mM drug. Moreover, WEB-2086 treatment of MDA-MB-231 decreased the active form of cathepsin D (lower band) and increased its inactive precursor protein (upper band) (Figure 4B); the latter was the only form present in low-invasive MCF-7 cells, irrespective of treatment.

### Molecular and biochemical changes induced by WEB-2086 on PBR, PAFR, ER*α*, PTEN and p53 in MCF-7 and MDA-MB-231 cells

**Figure 5:**
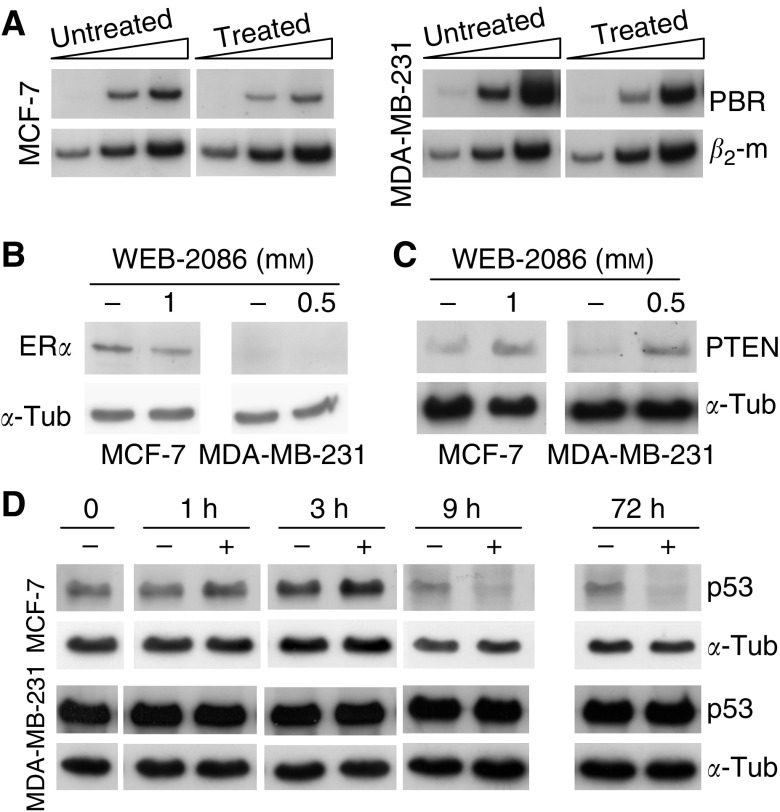
Effects of 1 and 0.5 mM WEB-2086 on PBR, ER*α*, PTEN and p53 expression in MCF-7 and MDA-MB-231 cells, respectively. (**A**) RT–PCR analysis of mRNA levels of PBR in cells treated for 5 days without (control) or with WEB-2086. Amplification of cDNA was carried out for an increasing number (25, 30 and 35) of cycles and *β*_2_-microglobulin (*β*_2_-m) was used as the reference gene; results were from a typical experiment out of three. Either untreated or treated MCF-7 or MDA-MB-231 cells were analysed by western blot for (**B**) ER*α* and (**C**) PTEN protein levels after 2 days of incubation with the drug, and for (**D**) p53 protein at 0, 1, 3, 9 and 72 h following WEB-2086 addition. *α*-tubulin was used as a loading control.

Drug effects on peripheral benzodiazepine receptor (PBR) mRNA levels in MCF-7 and MDA-MB-231 cells have been analysed; WEB-2086 was reported to bind these receptors (Svetlov *et al*, 1996) which are a hallmark of aggressive adenocarcinoma cells (Hardwick *et al*, 2001). After a 5-day treatment, PBR expression was downmodulated and, particularly, in highly invasive MDA-MB-231 cells (Figure 5A). Instead, PAFR expression was unaffected by the drug in both cell lines (data not shown).

The incubation of MCF-7 or MDA-MB-231 cells with WEB-2086 neither increased (actually, slightly decreased) nor restored ER*α* protein levels, respectively (Figure 5B). Importantly, an increase in PTEN protein relative to control was detected after a 2-day incubation with WEB-2086 in MCF-7 and, especially, in MDA-MB-231 cells (Figure 5C). Wild p53 levels in MCF-7 cells increased early in response to WEB-2086 with a peak at 3 h and then decreased progressively with time; instead, high levels of mutated p53 protein in MDA-MB-231 cells did not vary with treatment (Figure 5D).

## DISCUSSION

Studies on WEB-2086 have primarily been performed with leukaemia cells that were induced to differentiation and/or apoptosis. Here we proved that WEB-2086 might exert a wider anticancer activity being capable to decrease significantly proliferation also in human solid tumour cells of different histogenesis and with much higher efficacy than in normal cells. Then we focused on breast adenocarcinoma cells lines and showed that WEB-2086 can effectively inhibit *in vitro* cell growth and migration as well as relieve the differentiation block in ER*α*-positive MCF-7 and ER*α*-negative MDA-MB-231 cells. WEB-2086-induced growth arrest developed rapidly and led to a dose-dependent cell accumulation in G_0_–G_1_ phase with only a moderate activation of apoptosis. On a molar basis, differentiation efficacy of WEB-2086 was from 6- to 12-fold higher than that of butyrates and HMBA (Guilbaud *et al*, 1990).

Drug-induced changes in morphology and neutral lipid accumulation were more promptly elicited in ER*α*-positive MCF-7 cells while the decrease in mobility and active cathepsin D levels was especially observed in highly migrating ER*α*-negative MDA-MB-231 cells. Importantly, WEB-2086 effects were reversed upon drug removal to suggest that for potential therapeutic purposes carcinoma cells should receive the drug continuously as reported for other differentiation inducers such as butyrates (Guilbaud *et al*, 1990), TSA (Jang *et al*, 2004) and SAHA (Munster *et al*, 2001). Moreover, specific HDACi including valproic acid increased and/or reactivated ER*α* expression (Yang *et al*, 2000; Graziani *et al*, 2003) and sensitize both ER*α*-positive and ER*α*-negative breast cancers to treatment with tamoxifen (Jang *et al*, 2004). However, tamoxifen acts as an antioestrogen in breast, but as an oestrogen in the uterus, bone and cardiovascular system (Sommer and Fuqua, 2001) and its administration was associated with endometrial tumour development (Akhmedkhanov *et al*, 2001; Graziani *et al*, 2003). Noteworthy, WEB-2086 did neither increase nor restore ER*α* expression in MCF-7 or MDA-MB-231 cells, respectively.

WEB-2086 action mechanisms in breast cancer cells are still unclear but it is unfeasible that p53 could be involved as MDA-MB-231 cells express a mutated and inactive protein form. Moreover, the relatively high WEB-2086 amounts required to induce adenocarcinoma cell cytostasis and differentiation seem to rule out a relevant role of classical PBR and PAFR. This hypothesis is in keeping with other studies reporting that ligands of PBR like PK11195 and Ro 5-4864 (Carmel *et al*, 1999), and of PAFR such as SDZ 62-434 (Brunton and Workman, 1993) and CV 3988 (Bussolati *et al*, 2000) inhibited breast cancer cell proliferation at micromolar levels which are not consistent with high-affinity ligand-receptor binding. Importantly, WEB-2086 induced the upregulation of PTEN protein in MCF-7 and, especially, in MDA-MB-231 cells. PTEN plays a key role in several tumours including breast cancers by contrasting the activation of proto-oncogenic phosphatydilinositol 3′ kinase (PI3K)-Akt signalling pathway and also tumorigenesis (Pandolfi, 2004; Chen *et al*, 2005). Increase in PTEN levels and cell accumulation in G_0_–G_1_ phase as observed in treated breast cancer cells might be part of a more general mechanism common to other tumour cell types that, irrespective of their histology, were all sensitive to WEB-2086-induced growth arrest.

Overall, we demonstrated that WEB-2086 (a) induced a dose-dependent cytostasis in a panel of human cell lines from distinct solid tumours; (b) promoted morphological and functional differentiation in both ER*α*-positive MCF-7 and ER*α*-negative MDA-MB-231 cells through, possibly, PTEN upmodulation; (c) did not enhance/reactivate ER*α* expression; (d) synergized with HMBA. Two final comments might emphasize further the translational opportunities of WEB-2086. First, the drug proved to be relatively safe in animal models and humans (Adamus *et al*, 1989) and produced negligible alterations of clinical parameters when administered to thrombocytopenic patients (Lohmann *et al*, 1988; Giers *et al*, 1990). Second, WEB-2086 capability to abrogate the PAF-mediated signal will contribute to reduce growth, tumorigenic and metastatic potential of transformed cells, and impact also cancer microenvironment by contrasting neoangiogenesis (Bussolati *et al*, 2000).

## Figures and Tables

**Table 1:** Inhibition of cell growth by WEB-2086: IC_50_ values in cell lines from different human solid tumours and in control dermal fibroblasts

**Cell lines**	**IC_50_ (mM)^a^**
Breast adenocarcinoma (MCF-7)	0.65±0.09
Breast adenocarcinoma (MDA-MB-231)	0.41±0.07
Hepatocyte adenocarcinoma (Hep-G2)	0.78±0.07
Colon adenocarcinoma (HCT-8)	0.83±0.09
Fibrosarcoma (HT-1080)	0.38±0.04
Neuroblastoma (SH-SY5Y)	0.62±0.07
Dermal fibroblast (Control)	4.30±0.21

a Means values of four experiments.
